# Irisin: A Promising Diagnostic Biomarker at the Interface Between Systemic Inflammation and Skeletal Muscle Dysfunction in Haemodialysis Patients

**DOI:** 10.1111/nep.70102

**Published:** 2025-07-29

**Authors:** Rômulo D. Novaes, Robson E. Silva, Patrícia B. I. Justino, Mônica N. Barcelos, Thiago Donizeth Silva, Eliziária C. Santos, Fabiana C. S. A. Melo, Reggiani V. Gonçalves

**Affiliations:** ^1^ Programa de Pós‐Graduação Em Ciências Biológicas Instituto de Ciências Biomédicas, Universidade Federal de Alfenas Alfenas Minas Gerais Brazil; ^2^ Programa de Pós‐Graduação Em Biociências Aplicadas à Saúde (PPGB), Instituto de Ciências Biomédicas, Universidade Federal de Alfenas Alfenas Minas Gerais Brazil; ^3^ Programa de Pós‐Graduação Em Biologia Animal, Departamento de Biologia Animal Universidade Federal de Viçosa Viçosa Minas Gerais Brazil; ^4^ Faculdade de Medicina Universidade Federal de Alfenas Alfenas Minas Gerais Brazil; ^5^ Hospital Universitário Alzira Velano Alfenas Minas Gerais Brazil; ^6^ Faculdade de Medicina Universidade Federal de Viçosa (UFV) Viçosa Minas Gerais Brazil

**Keywords:** haemodialysis, irisin, kidney disease, muscle strength

## Abstract

Haemodialysis patients (HD) exhibit a pro‐inflammatory condition associated with catabolic and functional muscle disorders whose diagnosis and therapeutic management require more robust biomarkers. Thus, irisin relevance as a predictor of inflammation severity and muscle dysfunction in HD patients was investigated. From a prospective clinical study, irisin, tumour necrosis factor (TNF), C‐reactive protein (CRP), general metabolic markers, body composition, appendicular and respiratory muscle strength were measured at 1‐, 6‐, and 12‐month follow‐up. Body composition, haemoglobin, haematocrit, urea, creatinine, cholesterol, triglycerides, calcium, phosphorus, total protein and albumin levels remain stable in all time‐points investigated. TNF and CRP levels were upregulated, while irisin levels, handgrip strength (HGS), inspiratory (maximal inspiratory pressure—MIP) and expiratory muscle strength (maximal expiratory pressure—MEP) were attenuated in HD patients compared to healthy volunteers. Irisin levels were directly correlated with HGS, MIP, and MEP, partially explaining HGS (40%–54%), MIP (41%–61%) and MEP (40%–56%) variability. Irisin was inversely correlated with CRP and TNF levels, predicting 20%–25% CRP and 59%–63% TNF variability. Irisin exhibited good sensitivity in stratifying HD patients according to inflammatory and muscle strength status. Therefore, our findings indicated that systemic inflammation severity was associated with irisin depletion, which was a temporally stable predictor of muscle strength in HD patients. The discriminatory potential of irisin may be relevant for diagnosing HD patients with marked inflammatory stress and muscle dysfunction, refining the monitoring of CKD and dialysis therapy impact on inflammation and muscle strength, factors linked to functional capacity and risk of death in this population.

## 1 Introduction

Haemodialysis (HD) and kidney transplantation are the first‐choice treatments for end‐stage renal disease (ESRD) patients [1]. However, HD is not efficient in promoting a complete plasma clearance of uremic toxins, which accumulate and trigger intense metabolic disturbances orchestrated by systemic oxidative and inflammatory processes [1]. Accordingly, ESRD patients manifest a chronic uremic toxicity associated with metabolic acidosis, protein catabolism, and protein‐energy wasting (PEW), which are risk factors for premature death [2, 3].

There is evidence that PEW is linked to muscle wasting and sarcopenia, conditions associated with functional performance deterioration in HD patients [3, 4]. These disorders are aggravated by the upregulation of proinflammatory mediators (e.g., cytokines and chemokines) and inflammation severity [1, 5]. In this context, together with the intense protein catabolism, irisin depletion has been consistently demonstrated in HD patients [6, 7]. However, the consequences of this process are still poorly understood.

Irisin is predominantly produced by myocytes and plays important anti‐inflammatory and antioxidant effects, in addition to stimulating protein synthesis, mass gain, and energy metabolism in muscle tissues [6, 7]. Despite its immunomodulatory and myotrophic effects, the relationship between irisin, classical inflammatory markers (e.g., tumour necrosis factor [TNF] and C‐reactive protein [CRP]) and muscle function remains overlooked. Therefore, we conducted a prospective clinical study to evaluate the relevance and time‐dependent stability of irisin as a predictor of systemic inflammation, appendicular, and respiratory muscle strength in HD patients. Identifying robust biomarkers and predictive models that reveal the interrelationship between inflammatory effectors and muscle function may improve metabolic and functional monitoring of HD patients, in a continuous search for optimised diagnostic tools and treatment strategies.

## 2 Methods

### 2.1 Patients and Ethics

We conducted a longitudinal study in which sedentary patients with ESRD undergoing HD were evaluated for biochemical, immunological, anthropometric and muscle strength parameters at 1‐, 6‐ and 12‐month follow‐up. The absence of any type of physical activity was recorded in the medical records and confirmed with each patient in all reassessments. The entire population of adult patients (> 18 years) treated at the HD reference centre of our institution were eligible for the study. The following exclusion criteria were adopted: cognitive deficit, pregnancy, and change in dialysis modality in the last 3 months, haemodynamic instability, newly implanted catheters, neoplastic disease history, non‐agreement to participate in the study or to sign the informed consent form. The Institutional Ethics Committee in Human Research (protocol 1.717.736, December 02, 2023) approved this study, and all research volunteers signed the informed consent form.

### 2.2 Dialysis Characteristics and Study Protocol

All patients received a standardised haemodialysis protocol [8] as follows: 3–4 h sessions administered 3×/week on alternate days (Monday, Wednesday and Friday), 300–450 mL/min blood flow, and 500 mL/min dialysate flow. High‐flux and low‐flux polysulfone membranes, and sodium bicarbonate‐buffered dialysate were used. Dialysis adequacy was calculated by the K*t*/*V* method [8]. Blood samples were collected before the HD session, while anthropometric measurements were obtained after the session to mitigate water retention influence on body composition data [8].

### 2.3 Anthropometric Assessment

Body mass index (BMI) was calculated as BMI = body mass (kg)/body height (m)^2^ [9]. Lean mass and fat mass were calculated using the skinfold method based on the sum of skinfold thickness (suprailiac, biceps, triceps and subscapular) [8]. All measures were obtained in triplicate using a skinfold calliper with 10 g/mm^2^ pressure, 0–60 mm precision, and 1 mm scale (Lange, MI, USA) [8]. Arm circumference was measured with the limbs parallel to the trunk, adopting the midpoint between the scapula acromion and the olecranon as a reference. Adjusted‐arm muscle area was calculated from the results of triceps skinfold thickness and arm circumference [9].

### 2.4 Irisin and Tumour Necrosis Factor Immunoassay

Serum was obtained after blood centrifugation at 3000× *g* and 4°C for 15 min. Irisin was quantified using 96‐well ELISA kits, following the manufacturer's instructions (ThermoFisher Scientific, MA, USA). Tumour necrosis factor (TNF) was quantified by flow cytometry bead array (CBA) in a FACSVerse cytometer, according to the manufacturer's instructions (BD Biosciences, San Diego, CA, USA). Standard curves ranging from 20 to 5000 pg/mL were obtained using recombinant cytokines.

### 2.5 Metabolic Markers Biochemical Assay

Urea, creatinine, glucose, total cholesterol, HDL cholesterol, triglycerides, calcium, phosphorus, iron, and C‐reactive protein (CRP) were analysed in the same serum samples by spectrophotometry using commercial kits and the manufacturer's instructions (Invitro, MG, Brazil). Haemoglobin and haematocrit were quantified in blood samples using a haematological analyser (Sysmex, XE‐2100, SP, Brazil).

### 2.6 Hand Grip Strength and Respiratory Muscle Strength Tests

Handgrip strength (HGS) was measured using a mechanical Jamar dynamometer (Sammons Preston Inc., Warrenville, IL, USA) with an operational limit of 90 kgf, following the procedure recommended by the American Society of Hand Therapists [10]. Inspiratory and expiratory muscle strength were estimated by measuring the maximum inspiratory (MIP) and expiratory (MEP) pressures, as previously recommended [11]. The measurements were obtained using a MV‐150/300 manovacuometer with an operational limit of 300 cm H_2_O (Ger‐Ar, SP, Brazil).

### 2.7 Statistical Analysis

Data distribution was analysed by the D'Agostino‐Pearson method. Healthy volunteers (22 men and 18 women) with a similar age (54.19 ± 14.88) and BMI (23.55 ± 6.72) were included to estimate cut‐off points for irisin, TNF, CRP, and muscle strength. Chi‐squared or Fisher's exact tests were applied to categorical variables. Irisin and muscle strength data were compared at each time point analysed between HD patients and healthy volunteers using the Mann–Whitney U or Student's *t* tests, according to data distribution. One‐way ANOVA followed by the Student–Newman–Keuls method was used to compare all data collected at 1‐, 6‐, and 12‐month follow‐up. Data correlation was analysed by linear regression. The ROC curve (Receiver Operator Characteristic) was obtained from the stratification of inflammatory (CRP and TNF) and muscle strength (HSG, MIP and MEP) data above and below the median values for irisin (cutoff point). The discriminative potential of irisin was categorised according to the area under the ROC curve as follows: > 90%—excellent, > 80% to < 90%—good, > 70% to < 80%—regular, >60% to < 70%—weak, or < 60%—inadequate (Silva et al. 2019) [8]. Results with *p* ≤ 0.05 were considered statistically significant.

## 3 Results and Discussion

Fifty volunteers were included in the research. Six patients who received renal transplantation, three transferred to other hospitals and one with a change in dialysis protocol were excluded. Thus, 22 men and 18 women with similar age (men = 52.57 ± 16.11 vs. women = 55.09 ± 15.03 years, *p* > 0.05), time in dialysis (men 4.49 ± 2.98 vs. women = 4.05 ± 2.77 years, *p* > 0.05) and K*t*/*V* (≥ 1.2 [men *n* = 16, 40% vs. women *n* = 10, 32.5%, *p* > 0.05]) completed the 12‐month follow‐up. Comorbidities prevalence was similar between sexes, specifically diabetes mellitus alone (*n* = 30, 62.5%) or associated with systemic arterial hypertension (*n* = 8, 20.0%), systemic lupus erythematosus and hepatitis (*n* = 2 5%), *p* > 0.05. The anthropometric results (Table 1) indicated that at least 40% of patients presented overweight considering BMI ranging from 25 to 29.9 kg/m^2^ [9], ∑4ST fat mass > 18% for men and > 26% for women, as well as moderate malnutrition considering AC (70%–80% of the 50th percentile) and AAMA (> 5th and < 15th percentile) [9].

**TABLE 1:** Anthropometrical, biochemical, inflammatory, dialysis dose and muscle force parameters in haemodialysis patients (*n* = 40) evaluated at 1‐, 6‐ and 12‐month follow‐up.

Variables	1 month	6 months	12 months	*p* ^a^
Body mass, kg	67.20 ± 14.17	66.11 ± 13.09	66.95 ± 15.02	0.930
BMI, kg/m^2^	25.72 ± 4.98	24.94 ± 4.92	25.61 ± 5.86	0.774
Lean mass ∑4ST (%)	72.20 ± 11.76	71.18 ± 10.93	71.55 ± 10.88	0.918
Fat mass ∑4ST (%)	27.8 ± 11.76	27.82 ± 10.93	28.45 ± 10.88	0.918
AC, cm	28.18 ± 4.22	27.32 ± 4.14	28.46 ± 4.50	0.466
AAMA (cm^2^)	47.52 ± 13.26	46.66 ± 13.19	47.60 ± 14.02	0.942
Haemoglobin (g/dL)	11.94 ± 3.12	11.66 ± 2.50	11.80 ± 2.32	0.895
Haematocrit (%)	38.16 ± 8.13	36.22 ± 6.18	37.14 ± 6.70	0.230
Urea (mg/dL)	28.84 ± 10.44	30.18 ± 15.22	32.02 ± 18.45	0.639
Creatinine (mg/dL)	11.21 ± 2.18	12.36 ± 4.56	12.98 ± 5.30	0.169
Blood glucose (g/dL)	118.50 ± 44.40	126.15 ± 50.03	120.77 ± 42.90	0.746
Total cholesterol (mg/dL)	139.96 ± 25.12	145.17 ± 23.81	143.62 ± 20.72	0.591
HDL cholesterol (mg/dL)	41.30 ± 7.08	46.59 ± 18.42	44.22 ± 9.75	0.180
Triglycerides (mg/dL)	121.78 ± 40.36	129.15 ± 51.18	125.80 ± 46.13	0.774
Calcium (mg/dL)	8.05 ± 1.38	7.96 ± 1.25	8.57 ± 1.51	0.108
Phosphorus (mg/dL)	6.65 ± 2.71	6.52 ± 2.28	6.82 ± 3.21	0.888
Total proteins (g/dL)	7.04 ± 0.98	6.82 ± 0.79	7.17 ± 0.90	0.212
Albumin (g/dL)	3.56 ± 0.84	3.40 ± 0.91	3.79 ± 0.88	0.140
C‐reactive protein (mg/L)	9.55 ± 4.23	11.38 ± 4.14	10.25 ± 4.01	0.139
TNF (ng/mL)	0.21 ± 0.05	0.22 ± 0.05	0.23 ± 0.04	0.166
Irisin (ng/mL)	75.29 ± 12.13	69.62 ± 13.44	71.18 ± 10.56	0.100
K*t*/*V*	1.36 ± 0.30	1.34 ± 0.37	1.38 ± 0.35	0.871
HGS (kg)	22.64 ± 8.12	21.48 ± 7.87	21.91 ± 7.37	0.797
MIP (cm H_2_O)	62.21 ± 10.47	60.20 ± 10.11	60.90 ± 11.45	0.695
MEP (cm H_2_O)	84.60 ± 11.31	81.14 ± 10.47	82.89 ± 10.80	0.365

Abbreviations: ∑4ST, sum of four skinfolds thickness; AAMA, adjusted‐arm muscle area; AC, arm circumference; BMI, body mass index; HGS, Hand grip strength; K, urea clearance dialyzer; MEP, maximal expiratory pressure; MIP, maximal inspiratory pressure; *t*, treatment time; TNF, tumor necrosis factor; *V*, volume of distribution of urea..

^a^ Kruskal‐Wallis followed by Student–Newman–Keuls *post hoc* test.

Haematocrit (desirable/recommended = 33%–36% and frequently achieved = 31%–32%) and haemoglobin (10–11.5 g/dL) results (Table 1) were close to the conventional target range for haemodialysis patients [12, 13]. These findings rule out anaemia in most patients (80%), mitigating the impact of fatigue on muscle function, which is frequently observed at Hb < 9 mg/dL [13] and haematocrit < 29% [12]. Similarly, calcium and phosphorus levels were close to the reference values for these parameters (calcium = 8.4–9.5 mg/dL and phosphorus = 3.5–5.5 mg/dL) [14].

Total protein and albumin levels (Table 1) were also aligned with the results expected for HD patients [2]. Although the valuesidentified are within the normal range, HD patients present intense loss of amino acids (6–12 g), peptides and small proteins (1–3 g) during HD [2]. Thus, circulating protein levels are maintained at the expense of intense protein catabolism and muscle wasting [2], which are directly correlated with a decline in functional performance [3, 4] and increased mortality risk in these patients [2, 3].

Current evidence suggests an important relationship between protein catabolism and systemic inflammation in HD patients [1, 5], a characteristic identified in all evaluations considering significant differences (*p* < 0.001) in CRP and TNF levels (Table 1) compared to healthy volunteers (CRP = 0.21 ± 0.08 and TNF = 0.06 ± 0.01 ng/mL). High CRP and TNF levels contribute to oxinflammatory stress and vascular damage [5], which are associated with a risk of death up to 30× higher in HD patients compared to the general population [15]. In contrast, irisin levels (Table 1) were significantly reduced (*p* < 0.001) in HD patients compared with healthy volunteers (189.46 ± 20.72 ng/mL). This finding reinforces the evidence that irisin is down‐regulated in ESRD patients [7, 16]. Accordingly, irisin decline is apparently inversely correlated with systemic inflammation severity, protein catabolism, and muscle wasting in HD patients [7, 16].

In general, inadequate dialysis dose is linked to greater uremic toxins accumulation (e.g., urea, creatinine, and pro‐inflammatory cytokines) and systemic inflammation worsening [1]. Although the mean K*t*/*V* values indicated an adequate dialysis dose for the sample investigated (Table 1), 25%–30% of patients presented K*t*/*V* < 1.2 in at least one time‐point analysed, a characteristic potentially linked to systemic inflammation and protein catabolism worsening in these patients [1, 3]. Reduced K*t*/*V* is not an unusual finding, since it is directly influenced by protein and water intake, whose occasional variations significantly influence urea distribution and K*t*/*V* values [1].

Like K*t*/*V*, HGS, MIP and MEP results presented remarkable stability throughout all time‐points evaluated (Table 1). Although HGS was similar (*p* > 0.01), MIP and MEP were significantly reduced (*p* < 0.01) in HD patients compared to healthy volunteers (MIP = 75.16 ± 9.04 cm H_2_O and MEP = 92.44 ± 8.52 cm H_2_O). A relationship between appendicular and respiratory muscle strength has been demonstrated. However, there is insufficient evidence to support the hypothesis that respiratory muscle weakness may precede appendicular contractile dysfunction in haemodialysis patients, which requires further study. The relationship between appendicular and respiratory muscle strength has been demonstrated in previous studies [17, 18]. However, there is insufficient evidence to support the hypothesis that respiratory muscle weakness may precede appendicular contractile dysfunction in haemodialysis patients, which warrants further investigation. However, there is evidence that dietary modification/malnutrition and protein‐energy wasting (PEW) cause marked changes in body composition in HD patients [8]. Accordingly, muscle wasting has been directly associated with impaired functional capacity and increased morbimortality in ESRD patients [3, 4].

**FIGURE 1:**
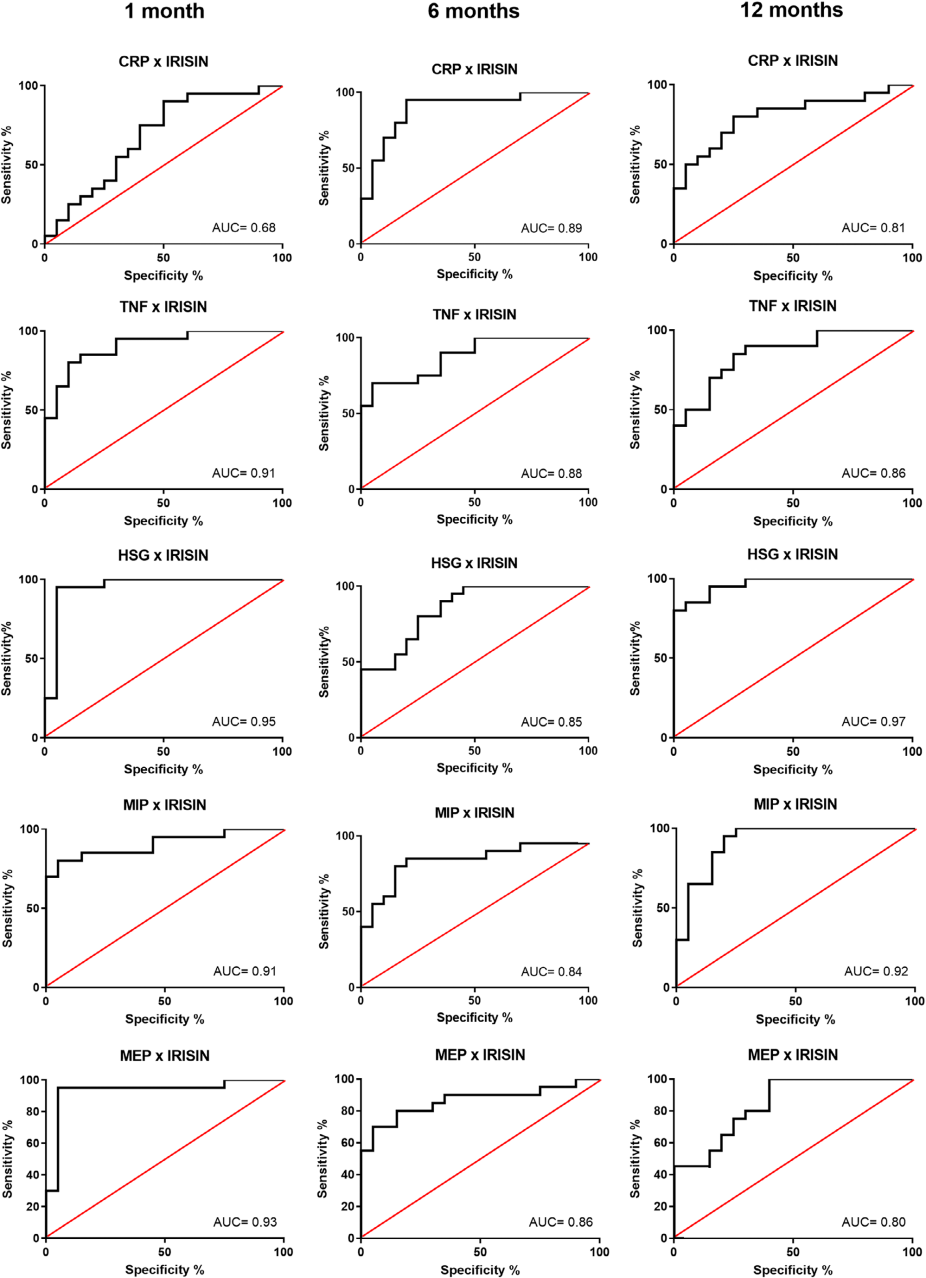
ROC (Receiver Operator Characteristic) curve of irisin levels in relation to inflammatory and muscle strength parameters in haemodialysis patients (*n* = 40) evaluated at 1‐, 6‐, and 12‐month follow‐up. AUC, area under the curve; CRP, C‐reactive protein; HSG, Hand grip strength; MEP, maximum expiratory pressure; MIP, maximum inspiratory pressure; TNF, tumour necrosis factor.

As muscle strength determinants are still poorly understood in HD patients, linear regression analysis allowed us to explore the relevance of irisin as an indicator of muscle contractile performance (Table 2). Interestingly, our findings revealed that circulating irisin levels can be used as predictors of muscle strength and systemic inflammation severity. Accordingly, irisin levels were directly correlated with muscle strength, being able to partially explain HGS (40%–54%), MIP (41%–61%) and MEP (40%–56%) variability. Furthermore, irisin was inversely correlated with systemic inflammation markers, being able to predict 20%–25% CRP and 59%–63% TNF variability (Table 2). In addition, the area under the ROC curve indicated that irisin stratification was a sensitive strategy for predicting inflammatory and muscle strength status, exhibiting marked temporal predictive stability over the 12‐month follow‐up. Accordingly, this predictive sensitivity ranged from 68%–89% for CRP, 85%–90% for TNF, 85%–96% for HSG, 83%–92% for MIP, and 80%–93% for MEP (*p* < 0.05) (Figure 1), indicating that irisin levels can exhibit a good or excellent discriminant potential to aid in the diagnosis of HD patients at risk of presenting inflammatory and muscle strength disorders.

**TABLE 2:** Linear regression models evaluating the correlation between irisin and handgrip strength, maximum inspiratory and expiratory pressures, C‐reactive protein and tumour necrosis factor circulating levels in haemodialysis patients (*n* = 40) evaluated at 1‐, 6‐ and 12‐months follow‐up.

Irisin (ng/mL)	1 month			6 months			12 months		
	*β*	*R* ^2^	*p*	*β*	*R* ^2^	*p*	*β*	*R* ^2^	*p*
× HGS (kg)	0.465	0.483	< 0.001	0.396	0.458	< 0.001	0.513	0.540	< 0.001
× MIP (cm H_2_O)	1.183	0.492	< 0.001	0.960	0.412	< 0.001	1.590	0.611	< 0.001
× MEP (cm H_2_O)	1.254	0.561	< 0.001	1.040	0.466	< 0.001	1.252	0.404	< 0.001
× CRP (mg/L)	−0.154	0.195	0.004	−0.149	0.232	0.002	−0.189	0.247	0.001
× TNF (ng/mL)	−0.003	0.587	< 0.001	−0.002	0.602	< 0.001	−0.003	0.628	< 0.001

*Note:* Statistical significance, *p* < 0.05.

Abbreviations: CRP, C‐reactive protein; HSG, Hand grip strength; MEP, maximum expiratory pressure; MIP, maximum inspiratory pressure; TNF, tumour necrosis factor.

Malnutrition, PEW and muscle wasting have been primarily associated with reduced irisin levels in HD patients [6, 7]. These disorders have also been associated with sarcopenia and reduced muscle performance [3, 6, 7]. However, the role of inflammation and protein catabolism on irisin depletion in ESRD patients remains overlooked. Accordingly, our results suggest for the first time that irisin may be more relevant in predicting respiratory muscle strength than HGS. Since this myokine is mainly secreted by myocytes, it is not possible to rule out a differential activation of PEW mechanisms in myocytes of appendicular and respiratory muscles, which could partially explain the variable predictive relevance of irisin. In addition to its myotrophic effect, irisin exerts marked anti‐inflammatory activity [6]. In this sense, irisin down‐regulation in chronic inflammatory conditions is coherent, especially considering that pro‐inflammatory responses activated in ESRD patients antagonise anti‐inflammatory mechanisms, culminating in the depletion of anti‐inflammatory effectors (e.g., IL‐4 and IL‐10) in HD patients [1]. Finally, there is evidence that inflammation triggers proteolytic mechanisms associated with PEW in ESRD patients [5]. In this sense, TNF is known to accelerate energy expenditure and stimulate proteolysis via ubiquitin‐proteasome and NF‐kB upregulation [5]. Thus, the chronic systemic inflammation may be a mechanism underlying irisin depletion in HD, a proposition that should be confirmed or refuted in further studies. On the other hand, our findings reinforce the role of irisin as an advantageous marker to estimate the relationship between inflammation and muscle dysfunction, especially considering that this molecule is mainly produced by skeletal muscle, which does not occur with molecules proposed as muscle function indicators whose biological sources are more abundant.

## Conflicts of Interest

The authors declare no conflicts of interest.

## Data Availability

The data that support the findings of this study are available from the corresponding author upon reasonable request.
